# Psychophysiological interaction analysis for the detection of stimulus‐specific networks in reflex epilepsy

**DOI:** 10.1002/epi4.12622

**Published:** 2022-07-09

**Authors:** Felix Zahnert, Marcus Belke, Jens Sommer, Julia Oesterle, Vincent Möschl, Christopher Nimsky, Susanne Knake, Katja Menzler

**Affiliations:** ^1^ Epilepsy Center Hesse, Department for Neurology University Hospital Marburg, Philipps University Marburg Marburg Germany; ^2^ LOEWE Center for Personalized Translational Epilepsy Research (Cepter) Goethe‐University Frankfurt Frankfurt Am Main Germany; ^3^ Core Facility Brainimaging, Faculty of Medicine University of Marburg Marburg Germany; ^4^ Department for Psychiatry Philipps‐University Marburg Marburg Germany; ^5^ Institute of Neuropathology, University Hospital Marburg, Philipps University Marburg Marburg Germany; ^6^ Department for Neurosurgery University Hospital Marburg, Philipps University Marburg Marburg Germany; ^7^ Center for Mind, Brain and Behavior (CMBB) Philipps‐University Marburg Marburg Germany

**Keywords:** connectivity, functional MRI, psychophysiological interaction analysis, reflex epilepsy, seizure

## Abstract

We report detailed functional MRI (fMRI) analyses in a patient with reflex seizures elicited by driving along a specific rural crossroad or by watching a video thereof. Semiology consisted of epigastric aura, followed by a sensory seizure of the left hand and sporadic automotor seizures. The right amygdala‐region (rh‐amygdala) was surgically and electroclinically confirmed as the epileptogenic zone. Presurgical task‐fMRI was performed, during which videos of the driving along that specific crossroad (IC), of another crossroad (NC) or noise were presented. Independent component analysis was conducted, and one component was used to aid in selection of a seed region within the rh‐amygdala for subsequent psychophysiological interaction analysis (PPI). Here, the following regions showed stronger connectivity with the rh‐amygdala seed during the IC condition compared to NC: right > left visual cortex, bilateral insulae, and right secondary somatosensory cortex (S2), potentially explaining epigastric aura and left somatosensory seizure semiology. Contralateral analyses did not reproduce these results. Overall, the ictogenic stimulus elicited enhanced connectivity of the epileptogenic rh‐amygdala with visual cortex and further regions of potential seizure spread (S2, insula) as a putative mechanism of ictogenesis. Our results highlight the potential of PPI in the analysis of stimulus‐dependent networks in patients with reflex epilepsies to gain insight into seizure generation.

## 1 INTRODUCTION

Reflex seizures are seizures that can be reproducibly elicited via a specific stimulus.^1^ Such stimuli can be complex in nature, and it is thought that subsequent recruitment of large neuronal masses can facilitate seizure generation.^2^, ^3^ In subjects with reading epilepsy and in musicogenic epilepsy, e.g., an increase in complexity or emotional content of stimuli can lead to a greater likelihood of subsequent seizures, potentially via recruitment of multiple brain regions.^2^, ^4^ Furthermore, highly specific visual stimuli with emotional content have been reported to trigger seizures.^5^ It has been hypothesized that aberrant cerebral connectivity may contribute to the generation of reflex seizures.^4^, ^6^

Overall, it seems that excitation of a cortical region via the stimulus can lead to synchronized recruitment of large neural masses via abnormal connectivity to further, potentially epileptogenic brain regions, ultimately culminating in a reflex seizure.^4^ Therefore, the study of reflex seizures can give rare insights into the neural substrates of seizure initiation.

In the present paper, we provide a detailed functional neuroimaging analysis on reflex seizure generation in a patient with reflex epilepsy and report a framework to study networks associated with epileptogenic stimuli.

## 2 METHODS

### 2.1 Subject

We report a 61‐year‐old male with right temporal lobe epilepsy (clinical characteristics: see Table 1).

**TABLE 1:** Clinical patient characteristics as obtained from patient history and clinical diagnostics

Patient history			
Seizures	First seizure	Seizure semiology	Seizure frequency
	FBTCS, 2 years prior to presentation to this clinic, age 59	Epigastric aura→focal aware somatosensory seizure of left hand (→head version to the left→FBTCS)	Two FBTCS overall. Persisting focal seizures. All seizures only as triggered by seeing a specific crossroad
Medication	At first presentation to this clinic: Levetiracetam 1500 mg/day; at time of surgery: Eslicarbazepine 1200 mg/day		
Risk factors for epilepsy	None		
Triggers	Seizures strictly tied to driving along a very specific rural crossroad		

Clinical diagnostics			
In‐patient Video‐EEG monitoring	Sharp waves	Seizures	Triggers
	Right temporal sharp‐waves (T8)	3 seizures: Two focal impaired awareness automotor seizures One focal aware seizure (epigastric aura) Seizure onset at electrode T8 (right temporal) in all cases	No photoparoxysmal response. **All seizures triggered by watching a video of the ictogenic crossroad on a mobile phone**
3 T‐MRI	Increased intensity (FLAIR and T2‐weighting) and volume of the right amygdala		
Lumbar puncture	<5 cells/μl, no autoantibodies		
Neuropathology	Diffuse neuronal heterotopia in the white matter of the right amygdala, hippocampal head and temporal pole		

Abbreviation: FBTCS, focal to bilateral tonic clonic seizure.

The patient described recurring seizures strictly tied to driving along a specific rural crossroad. The patient had taken a video of a drive along the “ictogenic” crossroad (IC), which was used for provocation of seizures during video‐EEG‐monitoring.

Video‐EEG‐monitoring yielded three seizures that were strictly tied to the patient watching the video of the crossroad on his mobile phone. A refractory reflex epilepsy with a right mesiotemporal seizure onset zone and amygdala enlargement on MRI was diagnosed.

Epilepsy surgery was performed, and the right amygdala, hippocampal head, and temporal pole were resected. Neuropathological assessment revealed diffuse neuronal heterotopia in the white matter of these regions (Figure S1). At the last follow‐up of 15 months post‐surgery, the patient has remained seizure‐free, even when passing the IC (ILAE 1A).Table 1. Clinical patient characteristics.

### 2.2 Data acquisition

The patient gave informed written consent for publication of clinical and neuroimaging data.

The patient underwent MRI scanning using a 3 T Siemens Magnetom Trio Scanner with a 12 channel headcoil. Structural MRI (3D‐T1‐MP‐RAGE) was acquired with 1 mm^3^ isotropic voxels (TR = 1.9 s, TE = 2.26 ms, inversion time = 900 ms, field of view of 256 × 256 mm, flip angle = 9°), parallel imaging (GRAPPA), and an acceleration factor of 2. Task‐based functional MRI was acquired with 2.5 mm^3^ isotropic voxels (480 volumes, TR = 1.2 s, TE = 30 ms, multiband acceleration factor = 3, field of view 210x210 mm, flip angle = 55°, echo spacing = 0.59 ms), and two fieldmaps were acquired for EPI distortion correction.

### 2.3 Task‐fMRI

Task‐fMRI was conducted in a block design during which the patient was presented with randomly interleaved sections of videos of either the specific ictogenic crossroad (IC), another crossroad, unbeknown to the patient (“neutral crossroad”, NC), and gaussian noise as a control condition, consisting of a random flicker of a dotted pattern (“snow”). Each block was presented 10 times and all blocks lasted 18 s. The patient received buttons for his right index‐ and middle fingers, respectively, and was asked to press the index finger button in case he experienced a seizure, and the middle finger button when he felt that a seizure was over (the latter was indicated unreliably and therefore seizure‐blocks, too, were given a duration of 18 s, see Appendix S1).

### 2.4 MRI analysis

Functional MRI processing was conducted using the FMRIB Software Library v6.0 (FSL, https://fsl.fmrib.ox.ac.uk/fsl/fslwiki/), and the structural image was processed via Freesurfer's recon‐all pipeline (http://surfer.nmr.mgh.harvard.edu/). Preprocessing of the functional image consisted of EPI distortion correction (FUGUE), motion correction (MCFLIRT), spatial smoothing with a gaussian kernel (7 mm FWHM), registration to the structural scan and to standard space (MNI152), and independent component analysis (MELODIC) for manual rejection of artifactual components. The number of components was estimated automatically within MELODIC^7^ (166 components).

A general linear mixed model was used (FSL FLAME, cluster corrected, cluster forming threshold z = 3.7, cluster p‐threshold = 0.001) with the regressors ‘IC, “NC”, “noise” and “seizure” to compute their respective contrasts (>mean) as well as the contrast IC > NC.

Subsequently, psychophysiological interaction analysis (PPI) was conducted,^8^ for which a seed (421.875 mm^3^ = 27 voxels) was placed within the epileptogenic right amygdala (rh‐amygdala). Seed placement was guided by one component from MELODIC (for details see Appendix S1, Figure S2). This allowed for selection of a seed within the amygdala while avoiding an entirely manual seed placement. The same PPI was also conducted (a) using the entire rh‐amygdala as a seed and (b) using homologous contralateral seeds.

Psychophysiological interaction analysis was conducted using IC > NC and the extracted seed‐timecourse as psychological and physiological variables to form an interaction term for analysis of stimulus‐dependent (stronger during IC than during NC) functional connectivity of this region. To ensure that the results of PPI reflected state‐dependent connectivity beyond task‐activation effects, the regressors IC + NC, noise, and seizure were included in the model as covariates of no interest. A cluster‐forming threshold of z = 3.1 and a cluster p‐threshold of *p* = .001 were applied. The BrainNet Viewer software was used for image visualization.^9^

## 3 RESULTS

**FIGURE 1:**
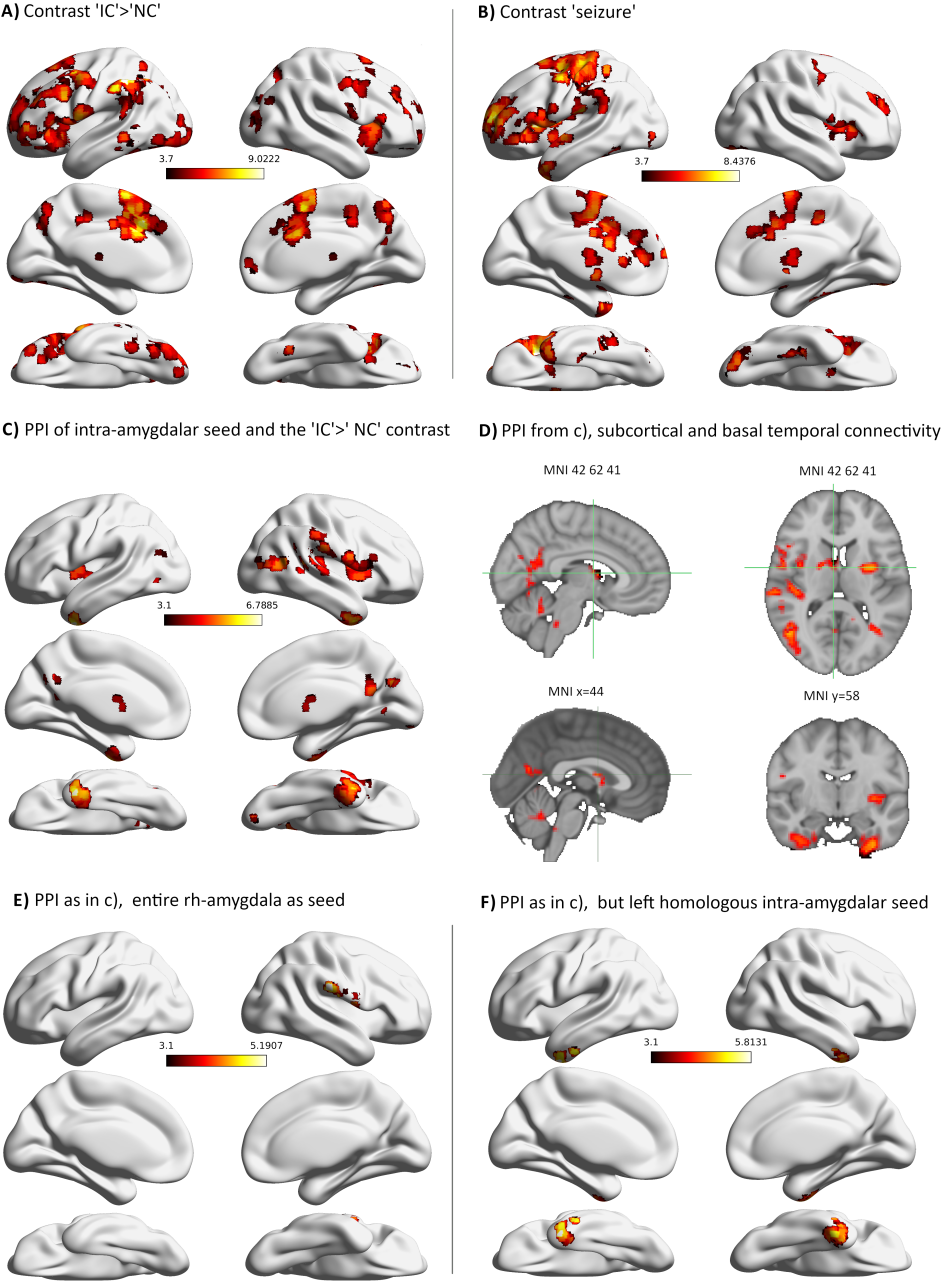
(A) Significant activations during the “ictogenic crossroad”>’neutral crossroad’ contrast and (B) during the “seizure” contrast (C) PPI of the timeseries of the seed region within the amgydala with the contrast IC > NC, i.e., functional connectivity of the amygdala‐seed stronger during seeing the “ictogenic” than during seeing the ‘neutral’ crossroad. (D) Depiction of subcortical and basal temporal connectivity during (C) the upper two panels depict small anterior thalamic connectivity, while the lower left panel shows the same cluster extending into the fornix as a correlate of the mesial subcortical cluster in (C). The lower right panel shows clusters in the ATLs. (E) Same analysis using the entire right amygdala as seed. (F) Results from PPI using a left‐sided, homologous seed (to that in (C)) within the non‐epileptogenic amygdala, and the IC > NC contrast

Task fMRI resulted in extensive activations that were stronger during IC compared to NC, including bilateral precuneus and cuneus, left>right posterior ventral temporal lobes, right anterior nucleus of the thalamus (ANT), bilateral insula, and predominantly left prefrontal cortex with peak activations in the left superior frontal gyrus (MNI 47‐65‐70) and left anterior cingulate cortex (MNI 47‐71‐53) (Figure 1A). No significant activations were detected for the NC > IC contrast.

During the scan, four seizures occurred, resulting in ictal activations in the left basal ATL, left cuneus, bilateral fusiform gyri and right lingual gyrus, bilateral prefrontal cortices and bilateral insulae (peak voxel: left insula, MNI 62–72‐41), and left precentral gyrus (Figure 1B). Interestingly, activations were present in bilateral frontal piriform cortices. An additional contrast for “IC with seizures”>’IC without seizures’ was computed, which yielded activations largely resembling those from the seizure contrast and is reported in the Appendix S1 (Figure S6).

Psychophysiological interaction analysis revealed a stronger state‐dependent relationship of the rh‐amygdala seed during the IC condition compared to NC with the following regions: right cuneus including right basal occipital cortex, bilateral right>left precuneus, bilateral basal anterior temporal lobes (ATLs, peak in left ATL, MNI 64–61‐16), bilateral right>left insulae, right supramarginal gyrus and right frontal and parietal operculum (Figure 1C). ATL subregions with significant interactions with the seed consisted of bilateral inferior temporal gyri, fusiform and parahippocampal gyri, and perirhinal cortex. Furthermore, significant task‐related connectivity was detected with the middle temporal visual area MT and a small, right‐sided cluster in the ANT.

Using the averaged timecourse of the entire right amygdala as physiological regressor, PPI with the IC > NC contrast reproduced right parietal opercular parts of the above network (Figure 1E), while PPI using the left amygdala as seed showed no significant interactions for this contrast. Using a smaller left‐sided amygdalar seed in a homologous location to the right‐sided analysis, task‐dependent connectivity (IC > NC) with only bilateral basal ATLs was detected (Figure 1F).

## 4 DISCUSSION

We demonstrated an increase in task‐dependent functional connectivity of the epileptogenic zone with (a) visual cortex and (b) secondary somatosensory cortex (S2) and the insula during watching of the ‘ictogenic’ crossroad as opposed to watching a random other intersection. We highlight for the first time the utility of PPI in delineating putative epileptogenic networks related to the ictogenic stimulus.

The ventral visual stream, which serves visual perception (the “what”‐pathway,^10^, ^11^, ^12^) is known to connect the visual cortex with temporal lobe structures such as the amygdala.^13^, ^14^ Psychophysiological interaction analysis confirmed task‐dependent connectivity of the epileptogenic amygdala to occipital and extra‐occipital visual areas, including areas attributed to the ventral visual stream (such as V2v/V3v^11^), but also to dorsal stream regions such as MT.^10^ Both streams are known to interact,^13^ and increased connectivity to the motion‐sensitive region MT^15^ seems plausible considering the present task.

Increased rh‐amygdala connectivity to S2 and to the anterior insula might constitute a pathway of seizure spread, as seizure semiology consisted in epigastric aura followed by sensory seizures of the left hand. Connectivity to somatosensory regions was confirmed even after averaging the signal across the entire right amygdala, while the left amygdala showed no task‐dependent connectivity to any region. Right rostral insula exhibited task‐dependent connectivity to the rh‐amygdala and showed activations during both the IC > NC contrast and the seizure contrast, hinting at its potential ictal involvement.

The temporal poles may facilitate ventral stream connectivity to the amygdala during emotional visual stimuli,^14^ which might explain the involvement of the ATLs in this network, as the patient reported anxiety in relation to the crossing. Therefore, connectivity of bilateral amygdala‐seeds to the ATLs may constitute physiological task‐dependent connectivity. We speculate that this ATL involvement might depict a correlate of an emotional stimulus leading to recruitment of a larger network that facilitates seizures.^4^ Similarly, bilateral perirhinal cortex, serving a function in object recognition and spatial orientation,^16^ exhibited state‐dependent connectivity with bilateral amygdala‐seeds. This may reflect increased physiological connectivity due to visual recognition of this emotionally connotated scene.

In PPI and task contrasts, a cluster was detected within the right ANT, which is known for its involvement in seizure propagation in focal epilepsies.^17^

Task‐activations for the seizure contrast yielded activations in bilateral piriform cortex as commonly seen in focal epilepsies.^16^ Insular activations overlapped with regions of increased task‐related connectivity, underlining the potential involvement of the insulae in the ictal network. Both the IC > NC contrast and the seizure contrast produced activations in the medial prefrontal cortex, which is commonly activated in fear conditioning, and which might be explained by the patient’s fearful reaction to the crossroad.^18^

### 4.1 Limitations

The main limitation of this study is its sample size of one subject due to the unique nature of the reflex epilepsy in this case. However, a plausible network associated with the ictogenic stimulus was detected that was robust to rigorous statistical thresholding, despite the inherently low statistical power of PPI.^19^ Testing of contralateral homologous regions was performed to confirm that stimulus‐dependent connectivity of the amygdala was selectively tied to the epileptogenic side. It is noteworthy that, despite motion correction, seizure‐associated motion may have impacted the quality of the scans.

It is unknown to what extent activations recorded during the seizure and IC > NC contrasts can be accounted for by visual recognition of the ictogenic, subjectively fear‐inducing crossroad. Unfortunately, it was unfeasible to control for this issue within the paradigm. Due to few seizures, which may have been unreliably detected (especially in duration), PPI was not conducted for the seizure contrast. Thus, it is not proven that the detected stimulus specific network is also seizure specific. Future studies combining EEG‐fMRI with PPI might overcome this issue.

## 5 CONCLUSION

In conclusion, PPI seems to be a promising tool to uncover epileptogenic networks otherwise undetected via canonical task‐fMRI activations in patients with reflex epilepsy. While PPI analyses have been used in the past to investigate cognitive compromise in epilepsy,^20^, ^21^ this is the first attempt to use PPI analyses to interrogate mechanisms of seizure propagation. We outlined a network underlying the ictogenic task, which consisted of visual regions and putative generators of seizure semiology connected to the epileptogenic amygdala.

## AUTHOR CONTRIBUTIONS

FZ‐ study conceptualization, data acquisition, data analysis and interpretation, manuscript writing and revision. MB‐ data acquisition and data analysis, manuscript revision. JS‐ data acquisition, data analysis, and manuscript revision. JO‐ data acquisition and manuscript revision. VM‐ analysis and interpretation of neuropathological data, manuscript revision. CN‐ data acquisition and manuscript revision. SK‐ study conceptualization, data interpretation, manuscript revision. KM‐ study conceptualization, data acquisition, manuscript revision.

## CONFLICT OF INTEREST

CN (unrelated to this research): CN served as a consultant for Brainlab. SK (unrelated to this research): SK received speaker´s honoraria from Arvelle, Bial, Epilog, Desitin, Precisis, UCB, and Zogenix. The remaining authors have no conflicts of interest to disclose.

## ETHICAL STATEMENT

We confirm that we have read the Journal’s position on issues involved in ethical publication and affirm that this report is consistent with those guidelines.

## Supporting information


FigureS1

## Data Availability

For data protection reasons, the imaging data reported in this study cannot be made available to the public.

## References

[1] Blume WT , Lüders HO , Mizrahi E , Tassinari C , van Emde Boas W , Engel J Jr . Glossary of descriptive terminology for ictal semiology: report of the ILAE task force on classification and terminology. Epilepsia. 2001;42:1212–8. 11580774 10.1046/j.1528-1157.2001.22001.x

[2] Striano S , Coppola A , del Gaudio L , Striano P . Reflex seizures and reflex epilepsies: old models for understanding mechanisms of epileptogenesis. Epilepsy Res. 2012;100:1–11. 22361339 10.1016/j.eplepsyres.2012.01.013

[3] Wilkins AJ , Bonanni P , Porciatti V , Guerrini R . Physiology of human photosensitivity. Epilepsia. 2004;45(suppl 1):7–13. 14706038 10.1111/j.0013-9580.2004.451009.x

[4] Koepp MJ , Caciagli L , Pressler RM , Lehnertz K , Beniczky S . Reflex seizures, traits, and epilepsies: from physiology to pathology. Lancet Neurol. 2016;15:92–105. 26627365 10.1016/S1474-4422(15)00219-7

[5] Mitchell W , Falconer M , Hill D . Epilepsy with fetishism relieved by temporal lobectomy. Lancet. 1954;264:626–30. 10.1016/s0140-6736(54)90404-3 13202455

[6] Vollmar C , O’Muircheartaigh J , Symms MR , Barker GJ , Thompson P , Kumari V , et al. Altered microstructural connectivity in juvenile myoclonic epilepsy: the missing link. Neurology. 2012;78:1555–9. 22551729 10.1212/WNL.0b013e3182563b44 PMC3348847

[7] Minka T . Automatic choice of dimensionality for PCA. In: Leen T , Dietterich T , Tresp V , editors. Advances in neural information processing systems. Cambridge, MA: MIT Press; 2000.

[8] Friston KJ , Buechel C , Fink GR , Morris J , Rolls E , Dolan RJ . Psychophysiological and modulatory interactions in neuroimaging. Neuroimage. 1997;6:218–29. 9344826 10.1006/nimg.1997.0291

[9] Xia M , Wang J , He Y . BrainNet viewer: a network visualization tool for human brain connectomics. PLoS One. 2013;8:e68910. 23861951 10.1371/journal.pone.0068910 PMC3701683

[10] Goodale MA , Milner A . Separate visual pathways for perception and action. Trends Neurosci. 1992;15:20–5. 1374953 10.1016/0166-2236(92)90344-8

[11] Kravitz DJ , Saleem KS , Baker CI , Ungerleider LG , Mishkin M . The ventral visual pathway: an expanded neural framework for the processing of object quality. Trends Cogn Sci. 2013;17:26–49. 23265839 10.1016/j.tics.2012.10.011 PMC3532569

[12] Wandell BA , Dumoulin SO , Brewer AA . Visual field maps in human cortex. Neuron. 2007;56:366–83. 17964252 10.1016/j.neuron.2007.10.012

[13] Milner AD . How do the two visual streams interact with each other? Exp Brain Res. 2017;235:1297–308. 28255843 10.1007/s00221-017-4917-4 PMC5380689

[14] Pehrs C , Zaki J , Schlochtermeier LH , Jacobs AM , Kuchinke L , Koelsch S . The temporal pole top‐down modulates the ventral visual stream during social cognition. Cereb Cortex. 2017;27:777–92. 26604273 10.1093/cercor/bhv226

[15] Galletti C , Fattori P . The dorsal visual stream revisited: stable circuits or dynamic pathways? Cortex. 2018;98:203–17. 28196647 10.1016/j.cortex.2017.01.009

[16] Vismer MS , Forcelli PA , Skopin MD , Gale K , Koubeissi MZ . The piriform, perirhinal, and entorhinal cortex in seizure generation. Front Neural Circuits. 2015;9:27. 26074779 10.3389/fncir.2015.00027 PMC4448038

[17] Child ND , Benarroch EE . Anterior nucleus of the thalamus: functional organization and clinical implications. Neurology. 2013;81:1869–76. 24142476 10.1212/01.wnl.0000436078.95856.56

[18] Etkin A , Egner T , Kalisch R . Emotional processing in anterior cingulate and medial prefrontal cortex. Trends Cogn Sci. 2011;15:85–93. 21167765 10.1016/j.tics.2010.11.004 PMC3035157

[19] O’Reilly JX , Woolrich MW , Behrens TEJ , Smith SM , Johansen‐Berg H . Tools of the trade: psychophysiological interactions and functional connectivity. Soc Cogn Affect Neurosci. 2012;7:604–9. 22569188 10.1093/scan/nss055 PMC3375893

[20] Tailby C , Kowalczyk MA , Jackson GD . Cognitive impairment in epilepsy: the role of reduced network flexibility. Ann Clin Transl Neurol. 2018;5:29–40. 29376090 10.1002/acn3.503 PMC5771327

[21] Trimmel K , van Graan AL , Caciagli L , Haag A , Koepp MJ , Thompson PJ , et al. Left temporal lobe language network connectivity in temporal lobe epilepsy. Brain. 2018;141:2406–18. 29939211 10.1093/brain/awy164

