# Supplementary material for: Psychophysiological interaction analysis for the detection of stimulus‐specific networks in reflex epilepsy
Source: Epilepsia Open. 2022 Jul 9;7(3):518–24. doi: 10.1002/epi4.12622 (PMC9436291; doi:10.1002/epi4.12622)
Supplement: Supplementary file 1 — FigureS1 [file EPI4-7-518-s001.docx]

**Supplementary material to the research article:**

*Psychophysiological interaction analysis for the detection of stimulus-specific networks in reflex epilepsy*

**Neuropathological findings**

Figure S1 depicts neuropathological findings in an exemplary location within the right anterior temporal lobe.


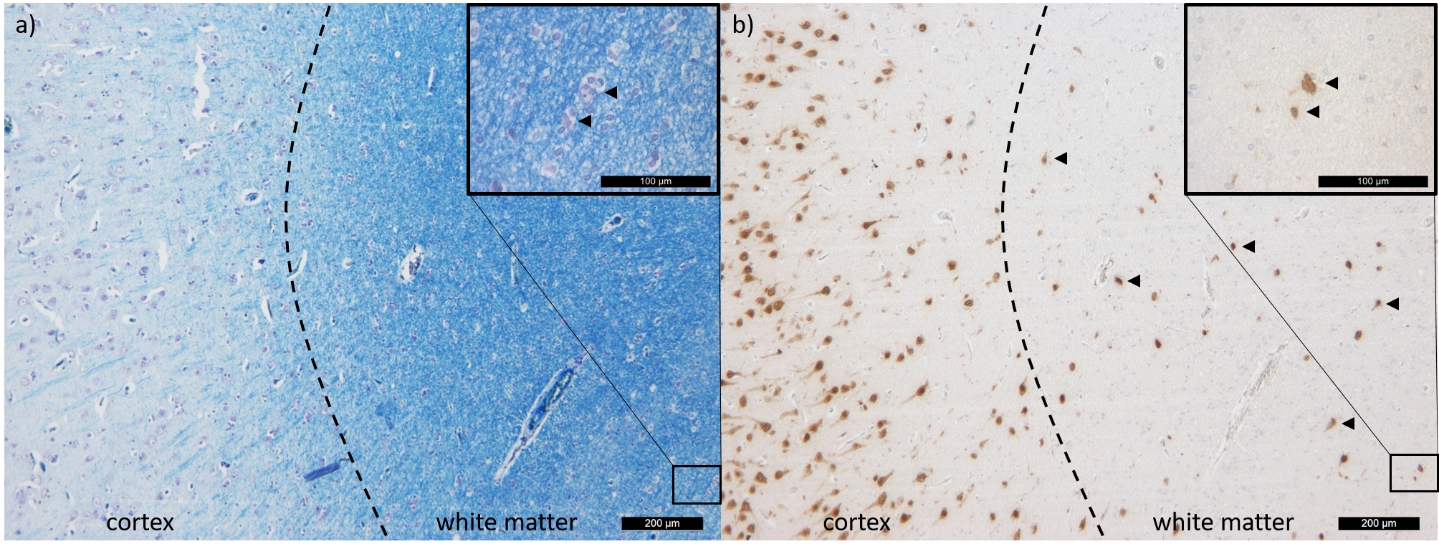


**Figure S1.** a) Kluver-Barrera stained slide of temporal pole tissue. The white matter shows an intense blue staining. b) Immunohistochemistry for NeuN of the same region. The arrowheads mark abundant neurons dispersed within the white matter indicative of diffuse neuronal heterotopia. Similar findings were observed in the white matter adjacent to the amygdala and the hippocampal head (not shown).

**Independent component of interest**

Independent component analysis was conducted for a) rejection of artifactual components and b) for data exploration. ICA revealed one component (out of 166 components estimated by MELODIC) that involved right occipital cortex, large parts of right sided ventral stream regions, as well as the right amygdala as the known epileptogenic zone. Neither further, similar components, nor left-sided correlates of this component were detected. Based on our hypothesis of a complex visual scene triggering seizures within the right amygdala via aberrant amygdalar connectivity with the visual system, this component was selected to aid in seed delineation for PPI. The component is depicted in Figure S2, and its timecourse can be appreciated in Figure S3. Here, interestingly, a relationship with the seizure regressor seems evident, in that the component consistently shows a drop in signal some seconds after the onset of a seizure.


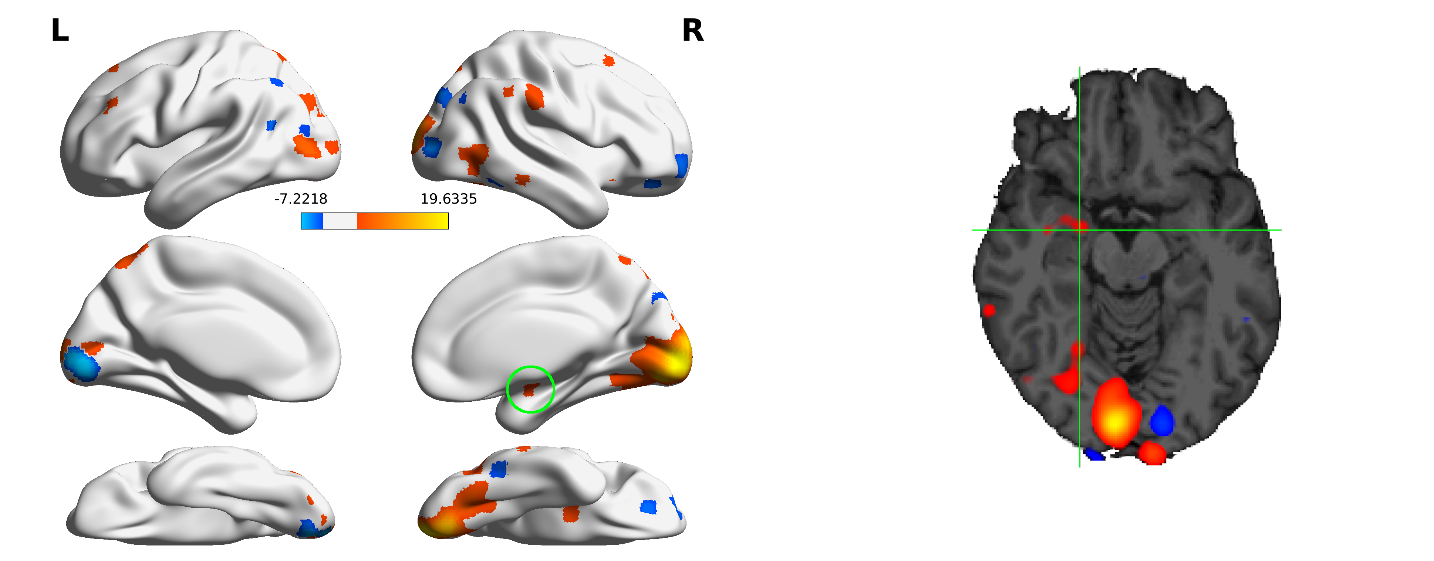


**Figure S2**. Independent component of interest. The green circle highlights the cluster within the right amygdala and piriform cortex, while the cursor in the right panel indicated the amygdalar peak voxel around which the 7.5mm³ seed was placed. Note that, besides visual regions, right secondary somatosensory cortex is also involved in this component.

**Timeseries of task-regressors, head motion and the independent component of interest**

Figure S3 depicts a pseudo design matrix showing task regressors as well as the timecourse of the independent component of interest and head motion parameters. All seizures occurred during clips of the ictogenic crossroad. The fourth seizure seemed to co-occur with increased head motion. Note that seizures had been treated as blocks of 18 seconds like the other EVs. While it was possible for the patient to denote seizure offsets via middle finger button presses, this was done too unreliably during the experiment for use in this analysis. Therefore, it is possible that the patient’s usually short seizures exceeded the respective task blocks of 18s.

The signal of the independent component seemed to positively fluctuate with the NC condition, while more negative deflections seemed to co-occur with the IC condition and especially during or shortly after “seizure” blocks.


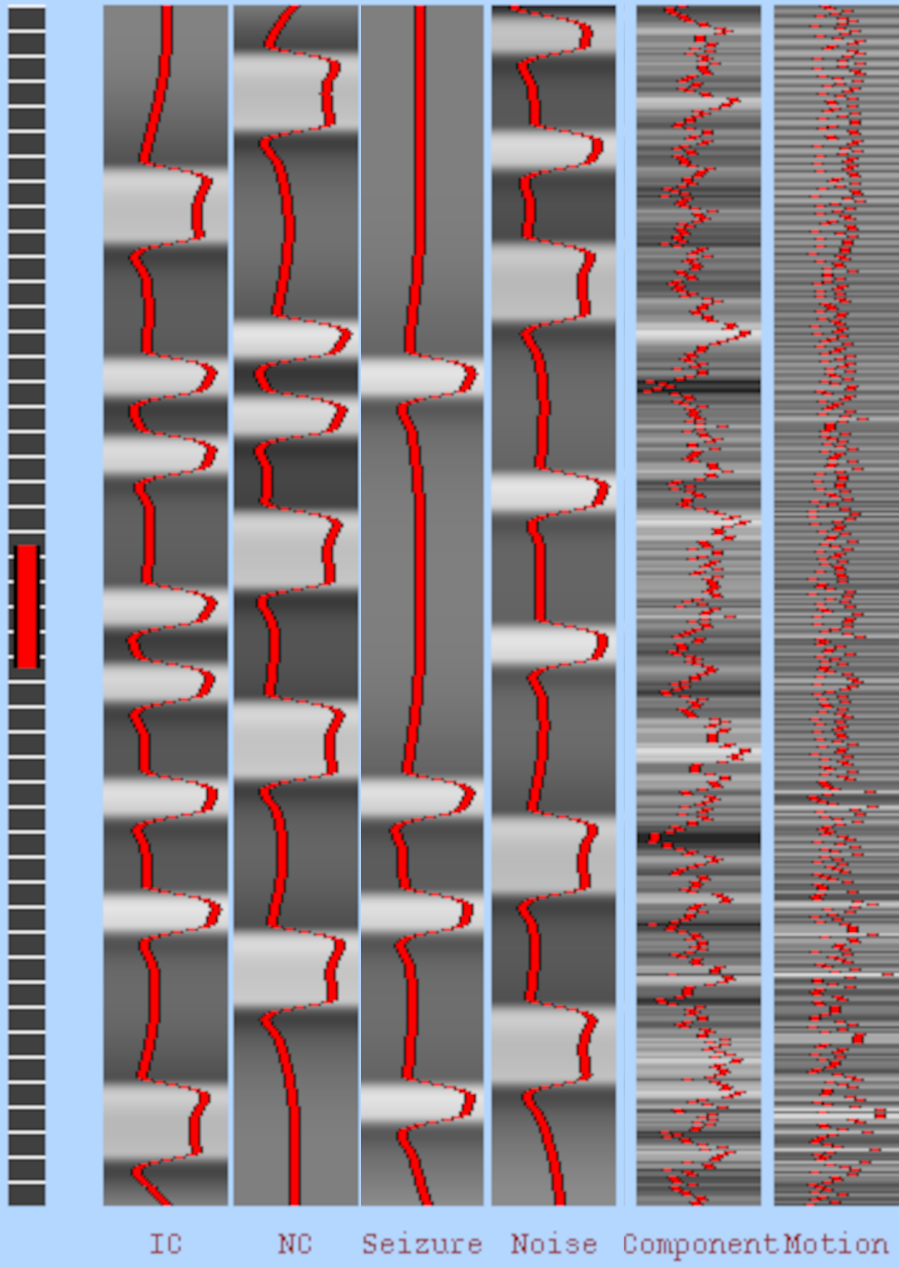


**Figure S3**. A pseudo design matrix is depicted showing the relevant task-regressors as well as the timecourse of the independent component used for seed selection and head motion parameters.

**Subcortical activations of task-contrasts**


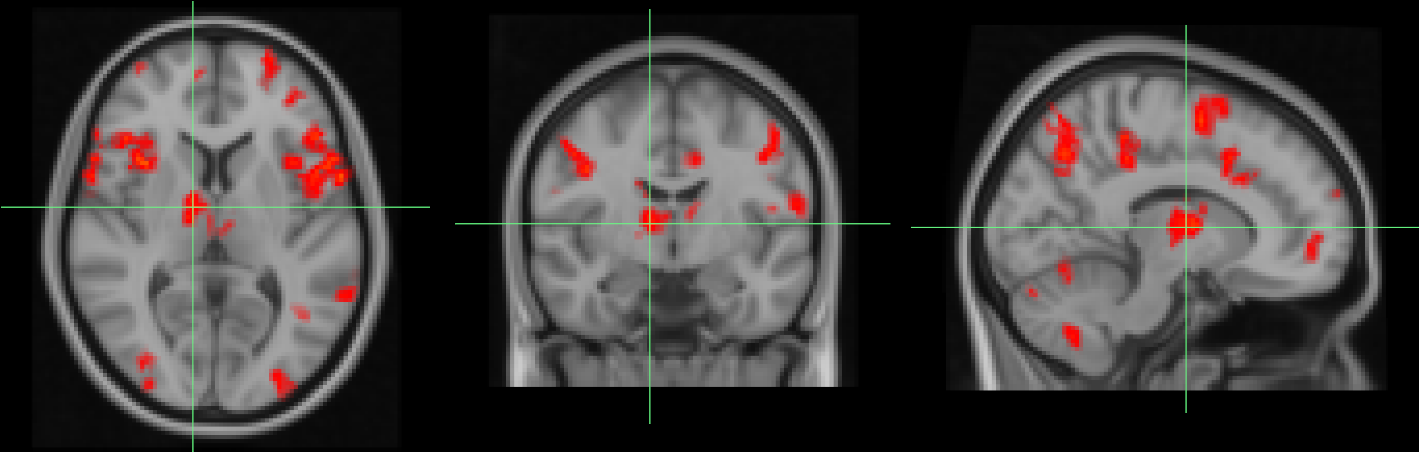


**Figure S4**. Task contrast IC>NC. A large cluster in the right anterior nucleus of the thalamus (ANT) is visible.


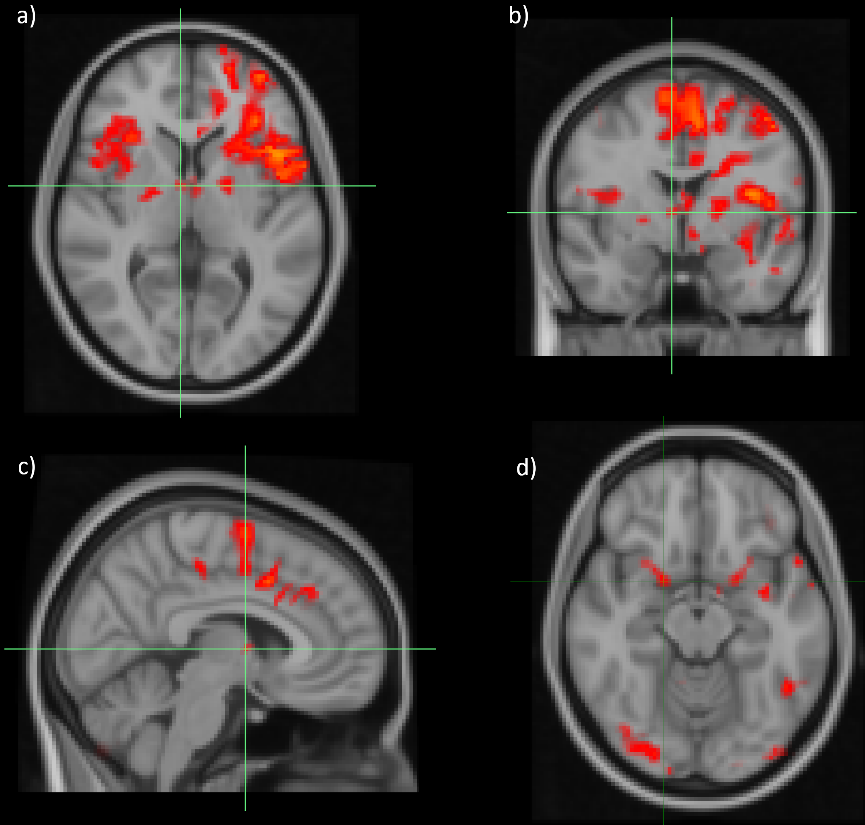


**Figure S5**. Task contrast for seizures. a)-c) show activations in the pallidum bilaterally, as well as small bilateral clusters in the ANT. d) Bilateral activations in the frontal piriform cortex can be observed.

**Activations for the contrast ‘IC with seizure>IC without seizure’**


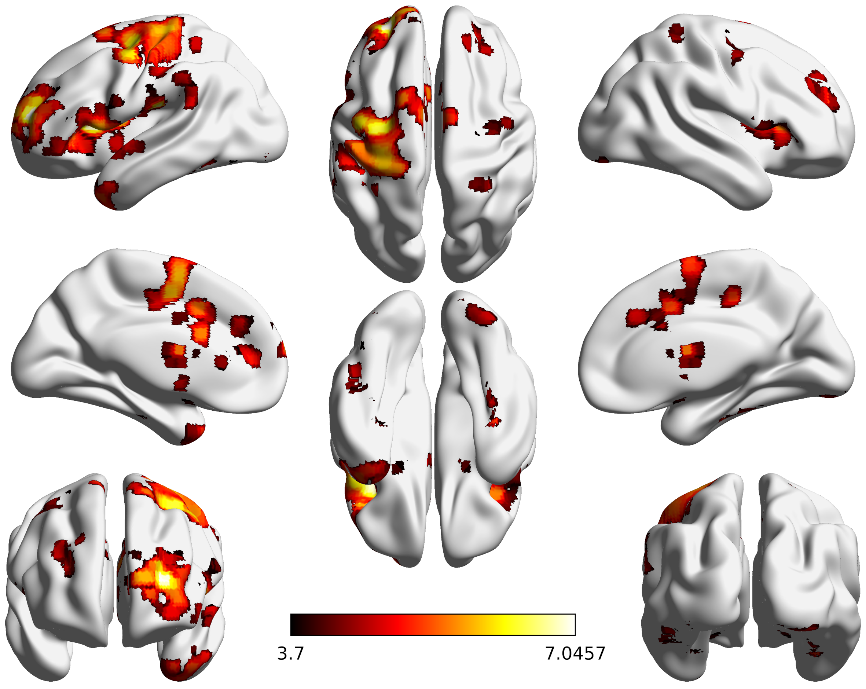


**Figure S6.** Task activations for the contrast ‘IC with seizure’ >IC without seizure’. Significant activations were detected in bilateral precentral gyri, most likely due to left-sided button presses. Activations for this contrast largely resembled the ‘Seizure’ contrast but were not identical. Opposed to the ‘seizure’ contrast, activations can be observed in right lateral parietal cortex, and left occipital activations are absent. Involvement of medial prefrontal regions (dorsal ACC, pre-SMA/SMA) may be related to negative emotions in relation to seizures and has been described in pavlovian fear conditioning (Etkin et al. 2011). The role of the observed predominant left sided activations remains unclear in relation to seizure semiology and location (e.g., no spread to the left hemisphere observed on EEG during focal seizures). Insular activations have also been observed during PPI and may be related to the perceived epigastric aura.

**Results from PPI using and alternative approach of motion correction**

As it may be argued that manual artifact rejection using MELODIC might be prone to investigator-related errors or bias, motion correction was alternatively applied by regressing out head motion parameters as confound EVs. This alternatively corrected data was only used to reproduce the main PPI analysis to demonstrate robustness of the obtained results to different motion correction approaches in the presence of in-scanner seizures.


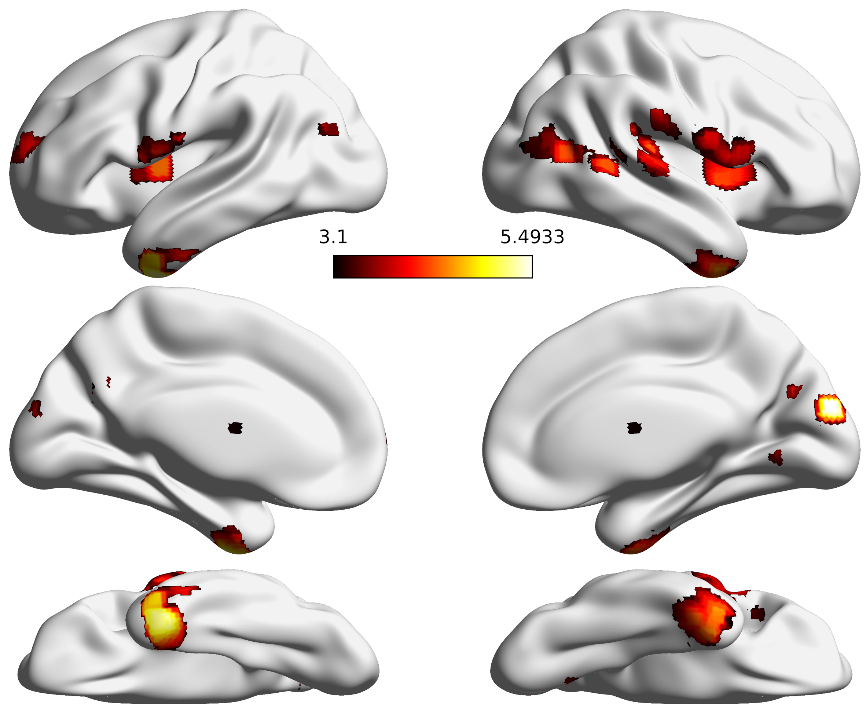


**Figure S7**. PPI using the IC>NC contrast and the timeseries of the 7.5 mm³ seed within the rh-amygdala as psychological and physiological regressors, while motion correction had been applied using regression of head-motion parameters instead of ICA. Results are nearly identical to those obtained in Figure 1c.

Publication bibliography

Etkin, Amit; Egner, Tobias; Kalisch, Raffael (2011): Emotional processing in anterior cingulate and medial prefrontal cortex. In *Trends in cognitive sciences* 15 (2), pp. 85–93. DOI: 10.1016/j.tics.2010.11.004.
